# Operation analysis of the tele-critical care service demonstrates value delivery, service adaptation over time, and distress among tele-providers

**DOI:** 10.3389/fmed.2022.883126

**Published:** 2022-08-05

**Authors:** Krzysztof Laudanski, Ann Marie Huffenberger, Michael J. Scott, Maria Williams, Justin Wain, Juliane Jablonski, C. William Hanson

**Affiliations:** ^1^Department of Anesthesiology and Critical Care, Hospital of the University of Pennsylvania, Philadelphia, PA, United States; ^2^Leonard Davis Institute for Healthcare Economics, Philadelphia, PA, United States; ^3^Department of Neurology, Hospital of the University of Pennsylvania, Philadelphia, PA, United States; ^4^Penn Medicine Center for Connected Care, Hospital of the University of Pennsylvania, Philadelphia, PA, United States; ^5^Campbell University School of Osteopathic Medicine, Lillington, NC, United States; ^6^University of Pennsylvania Health System, Philadelphia, PA, United States

**Keywords:** tele-ICU, tele-CCM, critical care, workflow, communication, intensive care unit, distress, implementation

## Abstract

**Background:**

Our study addresses the gaps in knowledge of the characterizations of operations by remote tele-critical care medicine (tele-CCM) service providers interacting with the bedside team. The duration of engagements, the evolution of the tele-CCM service over time, and the distress during interactions with the bedside team have not been characterized systematically. These characteristics are critical for planning the deployment of teleICU services and preventing burnout among remote teleICU providers.

**Methods:**

REDCap self-reported activity logs collected engagement duration, triggers (*emergency button, tele-CCM software platform, autonomous algorithm, asymmetrical communication platform, phone*), expediency, nature (*proactive rounding, predetermined task, response to medical needs)*, communication modes, and acceptance. Seven hospitals with 16 ICUs were overseen between 9/2020 and 9/2021 by teams consisting of telemedicine medical doctors (eMD), telemedicine registered nurses (eRN), and telemedicine respiratory therapists (eRT).

**Results:**

39,915 total engagements were registered. eMDs had a significantly higher percentage of *emergent* and *urgent* engagements (31.9%) vs. eRN (9.8%) or eRT (1.7%). The average *tele-CCM* intervention took 16.1 ± 10.39 min for eMD, 18.1 ± 16.23 for eRN, and 8.2 ± 4.98 min for eRT, significantly varied between engagement, and expediency, hospitals, and ICUs types. During the observation period, there was a shift in intervention triggers with an increase in *autonomous algorithmic ARDS detection* concomitant with predominant utilization of *asynchronous communication, phone* engagements, and the *tele-CCM module* of electronic medical records at the expense of the share of *proactive rounding*. eRT communicated more frequently with bedside staff (% MD = 37.8%; % RN = 36.8, % RT = 49.0%) but mostly with other eRTs. In contrast, the eMD communicated with all ICU stakeholders while the eRN communicated chiefly with other RN and house staff at the patient's bedside. The rate of distress reported by tele-CCM staff was 2% among all interactions, with the entity hospital being the dominant factor.

**Conclusions:**

Delivery of tele-CCM services has to be tailored to the specific beneficiary of tele-CCM services to optimize care delivery and minimize distress. In addition, the duration of the average intervention must be considered while creating an efficient workflow.

## Background

Telemedicine is a growing healthcare delivery modality with expansion accelerating after the onset of COVID-19 (1–3). This growth is particularly visible in tele-Critical Care Medicine (tele-CCM) due to the exacerbated pandemic needs and significant providers shortage (4, 5). However, the evolution of tele-CCM has been primarily organic and highly siloed with grass-root interactions between academic, federal, and private entities (1, 6). Until recently, growth has been dominated by a few industry partners and academic entities with relatively little diffusion of the protocols. Regulations and standards are mostly based on recommendations and expert opinions with limited support in evidence (5, 7–9). COVID-19 increased the stance of tele-CCM, but barriers cast uncertainty on post-pandemic development (5, 9, 10).

To date, task execution by the tele-CCM team has been studied relatively sparsely, even though the clinical workflows are critical for delivering high-reliable care (11). Placing unrealistic demands and expectations on providers will strain service delivery and increase the likelihood of burnout. There are gaps in knowledge regarding the frequency and duration of tele-ICU consultations. These metrics are critical for understanding the expected load on providers and service capacity.

The tele-ICU & bedside team dynamics are fundamental as communication is critical for care delivery. Fostering ingenuity of the bedside and tele-ICU staff is the strategy for finding ways of deploying TCC services, especially if the goals and visions of the tele-ICU deployment are unclear (12–14). The workflow must adapt interactions between tele- and bedside providers to address the needs most appropriate (11, 13–15). After launching the tele-CCM partnerships, the initial set of tasks will be modified to the most acceptable services. However, only few studies demonstrated a regionalization of the services (16–18). The execution of the primary goals will be influenced by the clinical and cultural specificity of the unit. Once the equilibrium between tele-CCM & bedside partnership is reached, maturation of service will take place (13). It is unknown how long the process of service evolution takes place.

The perception of tele-CCM performance has been predominantly studied from the bedside perspective (19–21). However, the remote team perception of the impact, professionalism, and potential distress secondary to interaction needs are critical for optimal interaction. Lack of understanding will inevitably affect the performance of the tele-CCM team and their engagement with the bedside. Inadequate interactions cause tension and emotional distress and contribute to burnout.

Here, we focused on characterizing the team dynamics of the tele-CCM staff providing care in large medical system. We aimed to characterize the execution of clinical tasks, their expediency, and the time needed for completion among three types of tele-CCM teams. We hypothesized that the execution of tasks would vary across the units depending on the local culture. We also studied team communication during task execution and their perception of acceptance of recommendations, emphasizing the distress caused by such interactions.

## Methods

### IRB consent

Considering this is a quality and improvement project, the study is exempted from the IRB's approval.

### Staffing model

The staff of the Penn E-lert™ consisted of 16 board-certified intensivists (eMD), 11 registered respiratory therapists (eRT), and 25 registered nurses (eRN), supported by our centralized telehealth coordinator team (eTHC).

The staffing model includes daytime and nighttime shift rotation equally staffed with three to four eRN, one eRT, and one eTHC who work 12-h continuous shifts. An unstructured sign-out to each other, noting the most urgent issues, occurs at 7 AM and 7 PM during shift changes. In addition, between 7 PM and 7 AM, one eMD joins the multidisciplinary tele-CCM team in the centralized hub.

The system supervised seven hospitals with 15 ICUs, each with different profiles comprising 295 beds (Supplementary Table 1). Staff within the ICUs comprises nurses (staffing ratio-1:2), RT (staffing ratio-1:8), and a variable combination of advanced practice providers, house staff, and medical attendings. All bedside medical attendings are board-certified in critical care.

### Workflow model

The Penn E-lert model has been established as a mixture of 33% reactive, 33% proactive, and 33% quality assurance but can adapt to the needs of the bedside team. The Penn E-lert mission is to provide highly reliable care within the health system. The tele-CCM team serves as the critical care consults hub for the multidisciplinary bedside staff who have the primary responsibility for patients. Penn E-lert actions could be triggered by a formalized review of patient medical records (*proactive rounding*). Several proactive tasks are well-defined to improve cooperativeness and homogeneity of responses (Supplementary Table 2). Tasks are also tailored to the provider's role in the system. eRTs and eRNs reported these tailored tasks as *aggregated tasks*. Prior video training assured common understanding by all staff.

The engagement was defined as any task related to patient care executed by a member of the team. An engagement by one member of the task force was independently executed from the action of other members. After initial reviews, eRNs and eRTs could escalate clinical issues to eMDs if they deemed the patient status severe enough to warrant intensivist review. eMDs would then review further and interact with bedside staff. The Penn E-lert and bedside staff could initiate interactions by either (1) engaging the emergency button installed in each ICU room or (2) contacting over the telephone by noting each other's mobile number as a member of the care team within the patient electronic medical record or, (3) engaging *via* an in-room audio-video system on the tele-CCM platform, or (4) using an asynchronous secure text message platform (22–24).

Routine tasks were addressed within 2 h, urgent tasks were addressed within 15 min, and emergent tasks demanded an immediate response. By default, proactive rounding was classified as a routine task unless other exceptional circumstances occurred.

Tasks were classified into six main categories: *clinical intervention* (any action to affect patient healthcare delivery), *quality and assurance* (review of the record or intervention to assure compliance with documentation), *safety* (intervention addressing potential harm), *education* (teaching provided to staff), *debrief* (review of complicated clinical situation, respectively), *recording* (documenting clinical situation per agreement with units), and *others* (unclassified). Any task could be classified with two of these descriptors.

Penn E-lert determined the need to communicate with the bedside team in response to engaging activity. In some cases, the intervention was terminated on review without requiring further external or internal communication. However, internal communication between members within Penn E-lert may still occur. For example, if communication with the bedside staff was needed, staff members connected *via* audio-video functions within the ICU room, telephone, secure text message, or consultation notes within the electronic medical record. At times, utilizing multiple communication avenues simultaneously were indicated within the survey.

At the end of an engagement, tasks' duration, complexity, and perceived difficulty spent on the task were recorded. Penn E-lert staff recorded their perception of the acceptance of the recommendation given during the interaction (*accepted, acknowledged, not accepted*) and if the engagement caused distress.

Data was collected between 9/1/2020 and 9/31/2021 using the final electronic REDCap survey database with staff inputting the data manually (Supplementary Material 1; Supplementary Figure 1) (25, 26). Each of the interventions is defined as “engagement” throughout the manuscript.

### Statistical analysis

The Shapiro-Wilk W test and distribution plots were used to test the normality of distribution variables. Homogeneity of variance was evaluated with Levene's test. Parametric variables were expressed as mean ± SD and compared using a *t*-Student test. For multiple parametric comparisons, ANOVA was used with *post-hoc* Turkey's case. χ^2^ was used to compare the frequencies between ordinary and nominal variables. A double-sided *p*-value < 0.05 was considered statistically significant for all tests. Statistical analyses were performed with Statistica 11.0 (StatSoft Inc., Tulsa, OK) or SPSS (IBM; Amonk; NY).

## Results

### Member's performance

**Figure 1:**
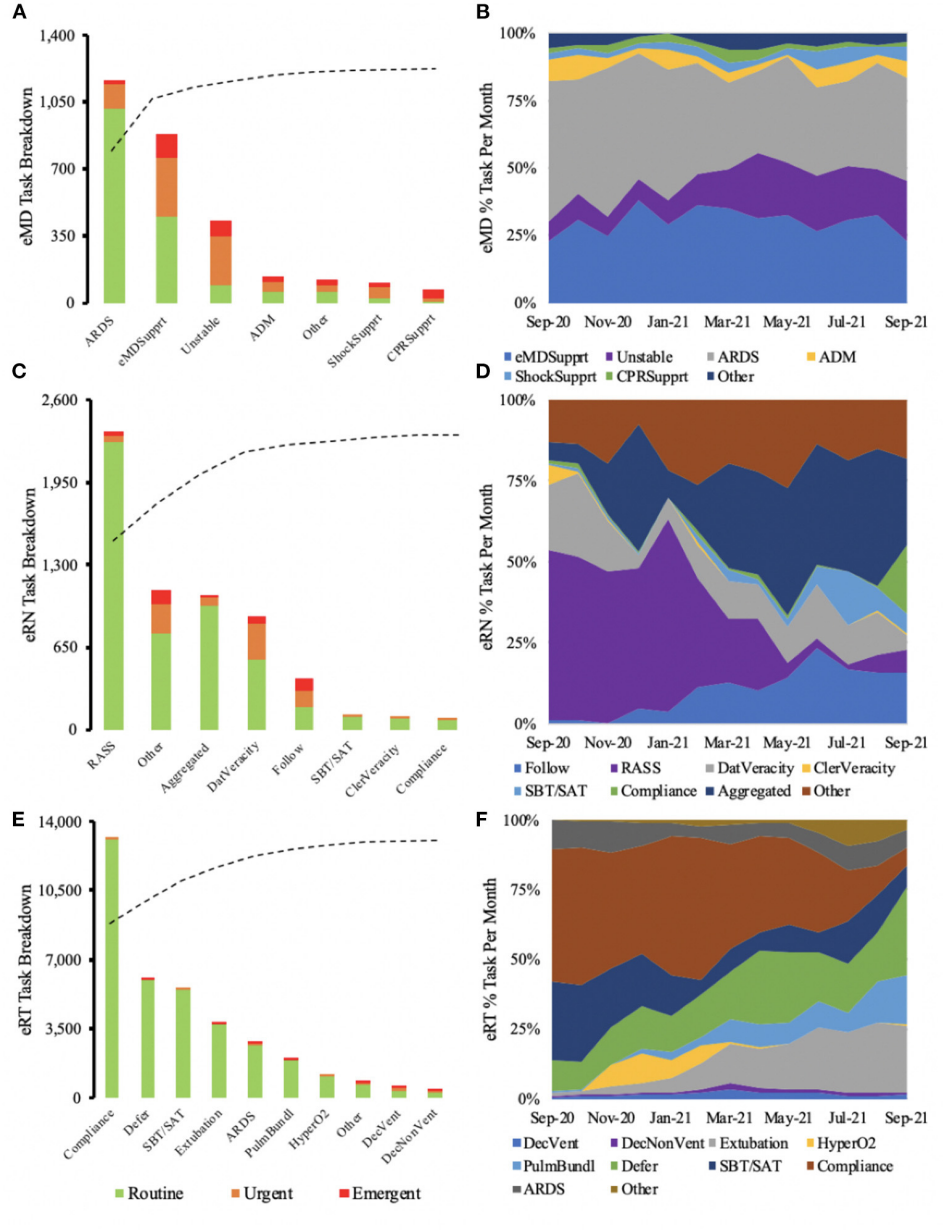
Majority of routine eMD tasks were associated with ARDS, while the majority of urgent and emergent interactions required eMD support **(A)**. Over the year, eMD tasks remained similar, with a slight increase in tasks involving unstable patients toward the end of the year **(B)**. eRN routine tasks were dominated by RASS **(C)**, but when observed over the year, RASS saw a steep decline toward the end of the year **(D)**. The majority of eRT routine tasks consisted of compliance **(E)**. As time progressed, there was a decrease in compliance and an increase in task deference **(F)**.

Over the observed time, 2,286 engagements were recorded by eMD [n_routine_ = 1,551 (67.8%); n_urgent_ = 559 (24.5%); n_emergent_ = 171 (7.5%); n_unclassified_ = 5 (0.2%). The majority of eMD tasks involved *ARDS* (40.5%), *intensivist support* (30.5%), and *unstable trends* (14.9%) (Figure 1A). eRNs executed 10,319 engagements [n_routine_ = 5,240 (50.8%); n_urgent_ = 795 (7.7%); n_emergent_ = 215 (2.1%); n_unlassified_ = 4,069 (39.4%)]. *RASS & delirium* (38.7%), *others* (18.1%) *and aggregated time* (17.2%) were most commonly executed eRN tasks (Figure 1C). Finally, 27,310 engagements were recorded by eRTs [n_routine_ = 26,820 (98.2%); n_urgent_ = 392 (1.4%); n_emergent_ = 79 (0.3%); n_unclassified_ = 19 (0.1%)] with the vast majority of tasks being routine and focusing on *compliance* (36.8%), *deferring* (16.7%) and *SBT/SAT* (15.4%) (Figure 1E).

31.9% of eMD engagements were deemed urgent or emergent, significantly higher than eRN (9.8%) and eRT (1.7%). On the other hand, eRT had significantly more routine tasks 98.2%, compared to the other specialties (Figures 1A,C,E; Table 1). However, variability existed between providers in each group (Supplementary Figure 2).

**Table 1:** Task breakdown per each specialty based on expectancy and total time spent in minutes.

**Staff**	**Task type**	***n***	**Total time (min)**	**Expediency**		
				**Routine**	**Urgent**	**Emergent**
eMD	eMD Supprt	877	1.670	448	311	118
	Unstable	428	10.355	95	251	82
	ARDS	1.165	15.460	1.016	131	18
	ADM	130	2.680	57	55	18
	ShockSupprt	99	2.530	26	56	17
	CPRSupprt	65	1.850	7	18	40
	Other	116	2.485	54	41	21
	eMD aggregated cases	2.880	37.030	1.703	863	314
eRN	Clinical follow-up	400	6.295	185	122	93
	RASS & delirium and sedation	2.340	29.920	2.268	57	15
	Investigating data veracity	893	15.235	550	285	58
	Clerical entry correction	94	1.690	92	2	0
	SBT/SAT	109	1.650	101	8	0
	Compliance	74	915	73	1	0
	Aggregated time DVT GI SBT	1.039	22.885	981	57	1
	Other	1.097	17.480	754	239	104
	eRN aggregated cases	6.046	96.070	5.004	771	271
eRT	DecVent	559	6.370	364	145	50
	DecNonVent	326	3.590	217	86	23
	Extubation	3.750	33.285	3.712	37	1
	HyperO2	1.098	6.525	1.089	9	0
	PulmBundl	1.898	17.075	1.895	2	1
	Defer	5.965	52.055	5.955	9	1
	SBT/SAT	5.488	34.715	5.448	40	0
	Compliance	13.105	98.275	13.077	28	0
	ARDS	2.713	20.530	2.680	32	1
	Other	747	13.095	697	38	12
	eRT aggregated cases	35.649	285.515	35.134	426	89

There was a little fluctuation in the different tasks performed for eMD across the observed year (Figure 1B). eRNs saw a significant reduction in *RASS & delirium* with the expansion of *aggregated tasks* toward the end of the year (Figure 1D). eRTs tasks switched from predominant *compliance* to *defer and extubation* (Figures 1E,F).

**Figure 2:**
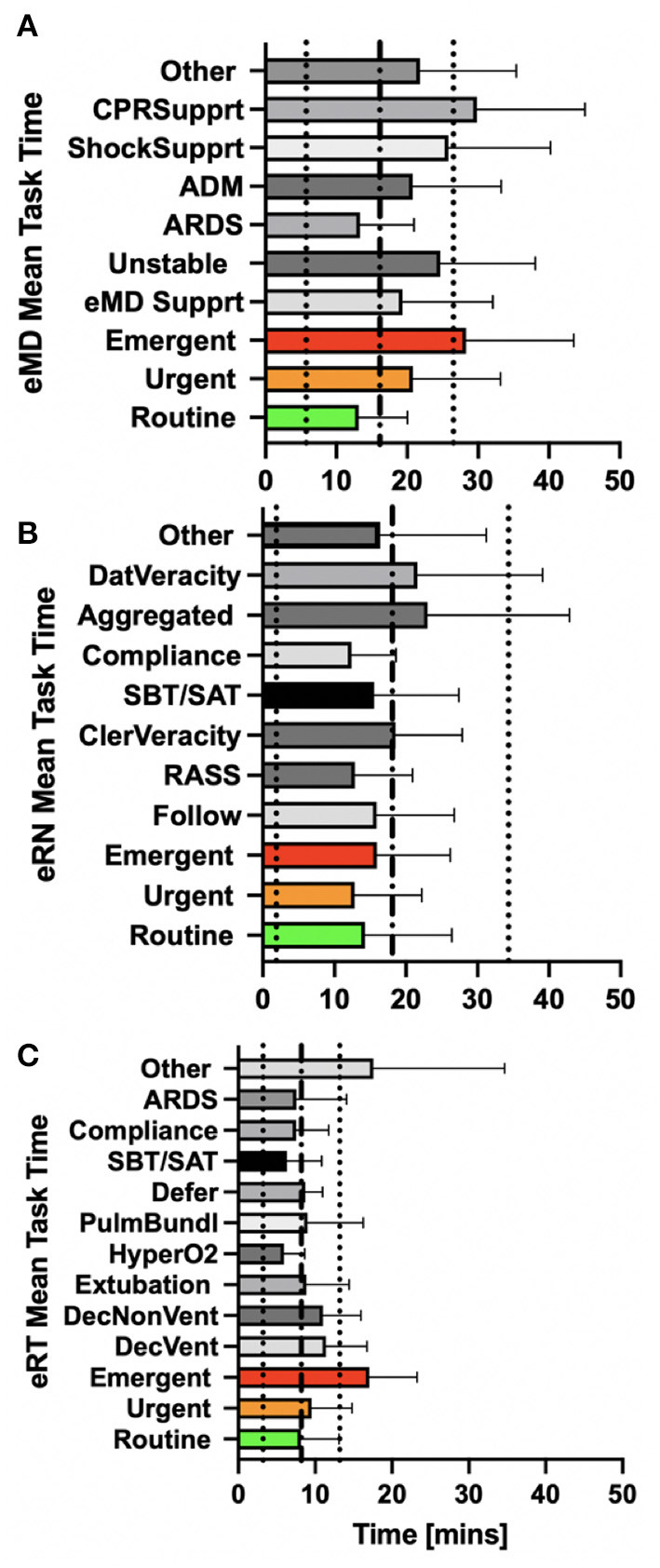
eMDs spent more time on tasks requiring CPR support followed by tasks that were deemed emergent **(A)**. eRNs spent the most time on aggregated patient tasks **(B)**. eRTs spent the most time on tasks described as other **(C)**.

There were significant differences in the time devoted to the tasks depending on the expediency across the different specialties (Figure 2). The average intervention took 16.13 ± 10.39 min for eMDs, 18.1 ± 16.23 for eRNs, and 8.19 ± 4.98 min for eRTs. Again, there were significant differences in time spent for different completing different assignments (Figure 2).

### Communication

**Figure 3:**
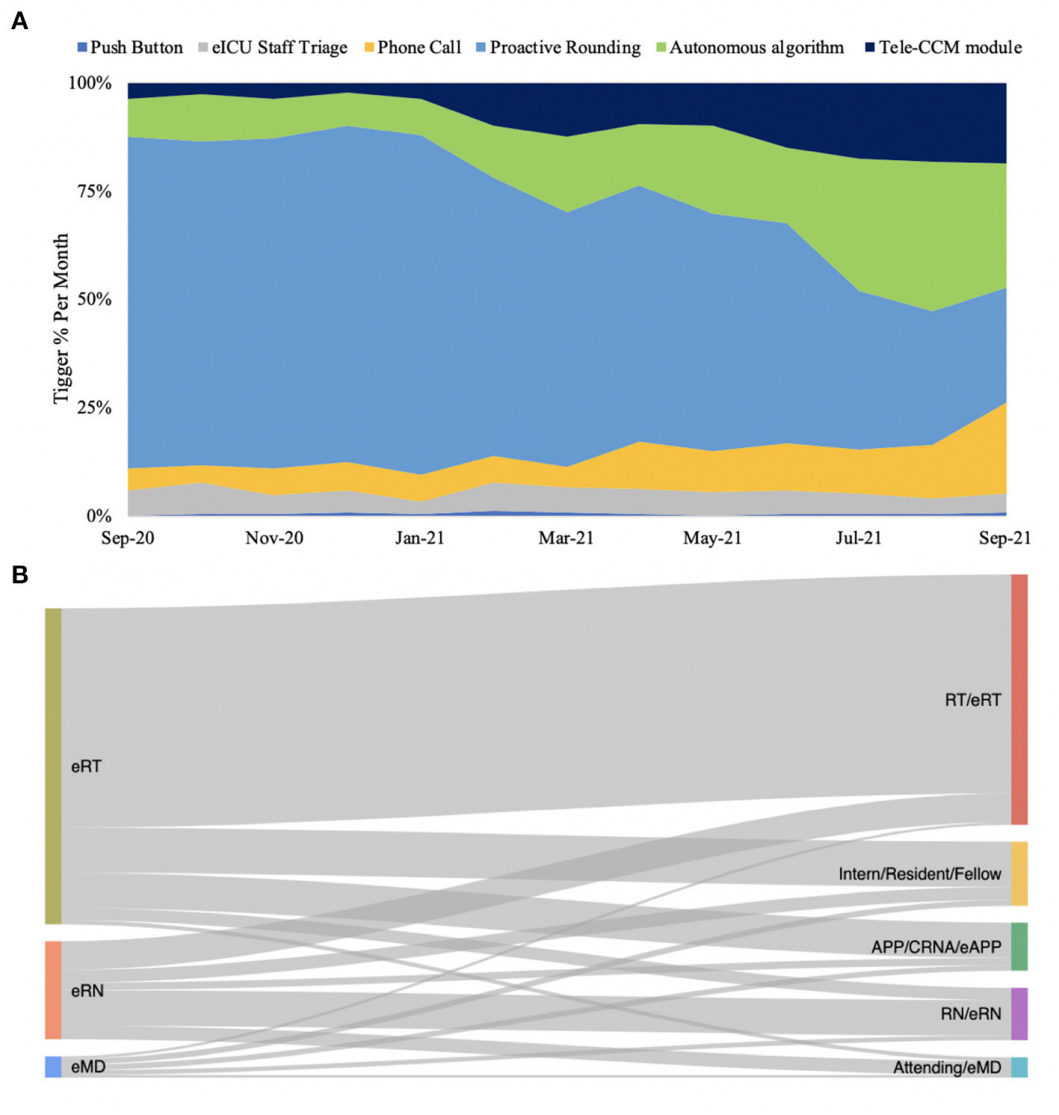
Push button was the greatest trigger for eMD, eRN and eRTs overall but there was a slight increase in Sniffer and Phone calls as triggers near the end of the year **(A)**, with the most significant amount of communication involving RT/eRTs **(B)**.

Initially, pro-active rounding triggers dominated with subsequent increases in ARDS autonomous algorithm triggers, telephone calls, secure text messages, and communications within the tele-CCM module in the electronic medical record (Figure 3A).

With eMD, eRN, and eRT engagements combined, no follow-up communication occurred in 70.1% of engagements, while communication did occur in 29.9% of recorded engagements. Among the Penn e-Lert staff, eRT communicated more frequently with bedside staff (% MD = 37.8%; % RN = 36.8, % RT = 49.0%). In cases when communication occurred between Penn E-lert and bedside staff, a significant variation between eMD, eRT, and eRN communication patterns were seen (Figure 3B; Supplementary Table 3). The eRNs communicated much more frequently amongst the Penn E-lert staff, while eMDs and eRTs communicated mostly with bedside staff (Figure 3B; Supplementary Table 3). eMDs communicated with various bedside stakeholders while eRNs engaged predominantly with bedside RNs.

### Regionalization of services

**Figure 4:**
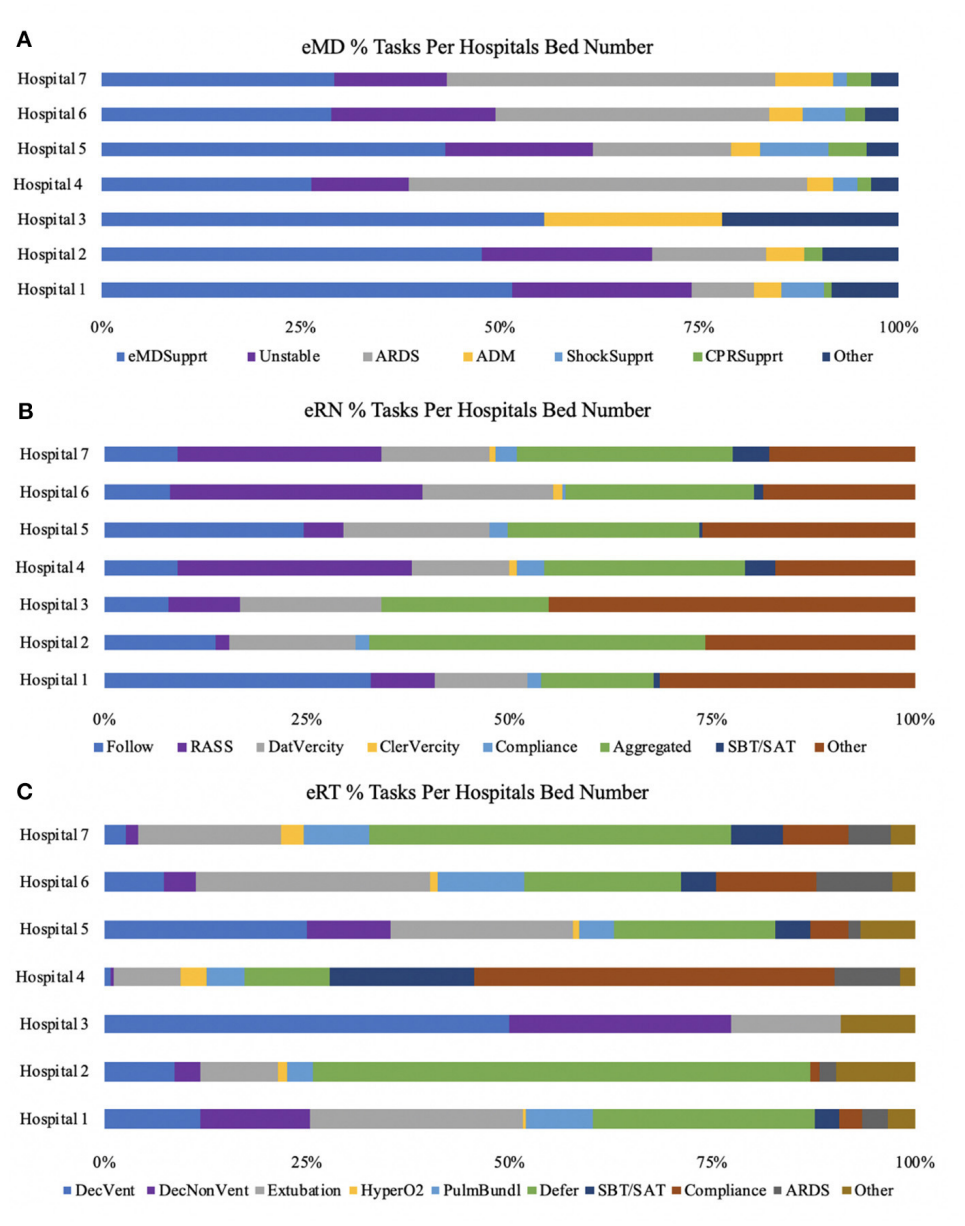
Percentages of tasks performed varied for each specialty based on the different hospitals. eMD support made up a large percentage of all hospital systems **(A)**. eRN tasks consisting of RASS was most significant across the larger hospital systems (hospital 4,6,7) **(B)**. eRTs had more task variability across the different hospital systems, but critical tasks were deference, extubation, and declining vent **(C)**.

There was a significant variance in the tasks delivered to each hospital and a significant variance across the different ICUs within each hospital (Figure 4, Supplementary Figure 5). Hospital 4 and 7 required more frequent *ARDS* interventions, while Hospital 1 utilized Penn E-lert eMDs (Figure 4A). *Clinical follow-up* dominated services provided to Hospital 4, Hospital 7, and Hospital 6, while *RASS & delirium* were the primary engagements within Hospital 5 in the case of eRTs. In the case of eRT, the task executed were remarkably heterogeneous, with *deferring* being at the highest incidence in Hospital 2 and Hospital 7. Hospital 3 predominantly requested *unstable vented and unstable non-vented*, while Hospital 4 requested *compliance*. Similar services were regionalized between units of the same profile regarding services provided by eMDs, eRTs, and eRNs (Supplementary Figure 5).

### Outcomes

The outcomes of the tele-CCM services can be measured in several ways. There was a total of 465 emergent and 1,746 urgent engagements. The push-button trigger was utilized in 132 and 21 of these engagements. These situations represent conditions where the level of the healthcare delivery was not adequate, triggering requests from the bedside for immediate support to advert clinical deterioration and thereby reducing mortality.

There were 2,641 aggregated tasks by eRNs that turned into 393 engagements consisting of checking alarms (*n* = 107, 27%), high risk extubation elements (*n* = 79, 20%), GI and DVT prophylaxis (*n* = 66, 7%), admissions (48, 12%), and other (*n* = 43, 11%). These engagements represent situations in which routine tasks turned into clinical interventions to avoid clinical deterioration and thereby reduce morbidity.

Finally, surveillance of compliance with hyperoxia avoidance and ARDS triggered by the autonomous algorithm resulted in modification of ventilator setting in and hyperoxia (*n* = 25, 6%), ARDS (*n* = 25, 6%). More importantly, Penn E-lert staff were able to identify ARDS-like patients earlier than the ARDS detection algorithm in 1,097 cases triggering the clinical adjustment in treatment when counting both eMD and eRT engagements.

Not surpassingly, COVID-19 patients were deferred more frequently to eRT service as compared to non-COVID patients. A total of 1,636 engagements involving COVID-19 patients were deferred to eRT staff resulting in PPE saving an amount of $41,848.88 and a total time of 272 h or roughly 11 days (27). A total of 3,027 engagements involving non-COVID-19 patients occurred, but we do not know how many were for the patients in isolation.

### Acceptance and self-reported distress rates during engagements

A significant number of the interactions were not specified in terms of acceptance as reported by Penn E-lert staff (eMD = 67.8% eRN = 64%; eRT= 54.4%). In cases when tele-CCM staff ranked the level of acceptance, eRT's reported excessive number of interactions described as *rejected* (Supplementary Table 4). However, eRNs had the highest ratio of interactions with “*reject*” as characterization while eMDs the lowest (% Rejected_eMD_ = 1%; % Rejected_eRN_ = 1.4%; % Rejected_eRT_ = 1.2%) (Supplementary Figure 3; Supplementary Table 4).

eRTs reported no distressful interactions, while eMDs had a distressing rate of 1.8% and eRNs had a rate of 1.9%. The different levels of interaction expediency had similar rates of distress, but certain hospital services had a significantly higher level of distressful interactions per bed (Supplementary Figure 4).

## Discussion

This is the first study detailing the workflow of the tele-CCM unit to foster a highly reliable service within the health system. The Tele-CCM team served as the critical care consultation hub for the multidisciplinary bedside staff who had primary responsibility for the patients. Our model utilized a pyramid model where eRTs and eRNs responded and analyzed the data provided by a computer system or a pre-defined list of tasks (28). If unable to address the problem, the case was escalated to an eMD. This model is optimal for utilizing expertise and skills by different providers.

We demonstrated that eRNs and eRTs effectively adopted the workflow to filter and assess the information provided by several clinical inputs to direct the work of eMD. Consequently, eRT and eRN tasks were dominated by routine tasks while eMDs responded to emergencies and urgent calls. eMD engagements were significantly longer. Across all healthcare providers, longer engagement times were seen during urgent and emergent tasks (29). This finding is consistent with the observation that emergencies are more engaging and complex. Also, an estimate of how long it takes to address the emerging clinical issues determines system operational capability. We determine that eRN or eMD can address around 36 interactions per 12-shift, a maximum of 16 min per interaction. In the case of eRTs, that capacity was significantly higher (~60 per shift). These numbers may guide staffing needs. No study has addressed the critical issue of engagement duration, and current estimates are opinion-based (30).

There was significant variability in tasks executed over time among eRN and eRT but not eMD. This may suggest that eMD reacted to eRN and eRT nudges generated during the proactive rounding. However, the eRN and eRT proactive rounding significantly fluctuated over time and may reflect adaptability to changing demand during waves of COVID-19 and adapting policies and priorities of the hospital. Emergent and urgent tasks tended to happen during night shifts, as has been published before (31).

We demonstrated a very high regionalization in the care delivered even between the units of similar profiles. Though this may represent a difference in the case mix, it may also reflect that different cultures and staffing models in these locations produced a unique set of demands that teleCCM staff could actively augment. Consequently, teleICU programs should be tailored to the needs of the bedside providers (32). In the case of our program, these needs were gradually adopted and modified by the staff as reflected in the change from proactive tasks to engagements triggered by specific inputs (tele-CCM module, automated alarms). The phenomenon of service customization was frequently suggested but rarely quantified (33). Remarkably, teleCCM services evolved, suggesting that the remote service providers adapted their behavior based on their needs (34). The importance of this observation allows determining how long it takes for teleCCM service to mature. Furthermore, we demonstrated that staff could find novel, or more efficient ways, to utilize the system and provide more accurate delivery *via* organic and grassroots processes. This study provides an indirect suggestion supporting this observation (35).

We demonstrated that most tasks did not trigger communication outside tele-CCM providers. This may reflect a high degree of triggered non-actionable alerts, a common healthcare problem. Additionally, the Penn E-lert team often deemed the engagements undertaken by the bedside team to be appropriate (36). However, the exact nature of engagements without communication remains unclear.

As mentioned above, we captured a high degree of internal collaboration within the remote site, while a minority of these cases resulted in interaction with bedside staff. These interactions occurred *via* all available communication channels with the variability of communication modes among eRN and eMD based on bedside stakeholders. In contrast, eRT collaborated mostly with RT at the bedside. These differences may reflect the high burden of protocolized tasks for eRTs compared to the tasks of MDs and eRNs. As a result, their workflow may be less protocolized and require staff to address unique problems more frequently.

The quantification of service benefits was demonstrated on several levels. Earlier recognition of ARDS and subsequent less frequent emergence of ARDS clearly demonstrate a measurable effect on clinical care (37). A similar actuarial calculation can be done for push-button situations, missed deep venous thrombosis prophylaxis, and stress ulcer prophylaxis. These interactions avert clinical deterioration and thereby reduce morbidity (38). Implementation of eRT, eRN, and eMD instead of bedside staff reduced PPE use and staff exposure to COVID-19 (4).

The acceptance rate for the recommendation varied considerably between the hospitals and units. Some hospitals implemented Penn E-lert significantly longer than others. The duration of engagement fosters trust and acceptance of recommendation. However, we did not account for the nature of interactions between bedside and remote teams as well as the acceptance of the technology (39).

Finally, we were able to quantify the rate of distress during engagements among the remote ICU staff at roughly ~2%. In a very limited *post-hoc* interview, the primary drivers were the lack of control and professionalism in conversations (40). Interestingly, a dominant factor in the frequency of stressful interactions was not the role of stakeholders but the entity interacted (41). Again, this may reflect challenges with the early adoption of telemedicine within certain hospitals (38).

The study has a couple of limitations. First, the data was entered by Penn E-lert staff voluntarily. We estimated that several interactions were not entered, especially those considered routine. This may account for significant differences in the type and volume of entries by different providers. We estimate that 30% of entries are most likely missing from the survey. Although several tasks were highly standardized and consistent, and educational materials were provided to reinforce the data collection definitions and expectations over time, there is a possibility of a different interpretation. High variability between providers in recorded numbers of entries may results in over-representation of a particular type of attending vs. those who tend to file less. Staff estimated time without corroboration with more independent measures. The service demand may change based on patient severity scores, but this data was not accessible during this analysis. Finally, REDCap survey tool was insufficient in capturing all the tasks delivered if they were not pre-programmed.

## Conclusions

We quantified the interactions of the tele-CCM staff in terms of their nature, duration, expediency, and value. High regionalization of service delivery and distress were observed, suggesting that delivery of tele-CCM services has to be tailored to the needs of the specific beneficiary of tele-CCM services.

## Data availability statement

The raw data supporting the conclusions of this article will be made available by the authors, without undue reservation.

## Ethics statement

Considering this is a quality and improvement project, the study is exempted from IRB's approval.

## Author contributions

KL: conceptualization, data collection, manuscript writing, and reviewing. MS, AH, MW, and JJ: execution and manuscript reviewing. JW: data visualization and manuscript reviewing. CH: execution, funds acquisition, and manuscript reviewing. All authors reviewed the final version of the manuscript and agreed to its publication.

## Conflict of interest

The authors declare that the research was conducted in the absence of any commercial or financial relationships that could be construed as a potential conflict of interest.

## Publisher's note

All claims expressed in this article are solely those of the authors and do not necessarily represent those of their affiliated organizations, or those of the publisher, the editors and the reviewers. Any product that may be evaluated in this article, or claim that may be made by its manufacturer, is not guaranteed or endorsed by the publisher.

## Abbreviations

eMD: telemedicine medical doctor
eRN: telemedicine registered nurse
eRT: telemedicine respiratory therapist
eTHC: telehealth coordinator team
tele-CCM: tele-critical care medicine
TCC: telemedicine critical care
ARDS: acute respiratory distress syndrome
ADM: admissions
ShockSupprt: shock support
CPRSupprt: cardiopulmonary resuscitation support
SBT/SAT: spontaneous breathing trial/spontaneous awakening trial
DecVent: intubated patient status declining
DecNon Vent: non-intubated patient status declining
HyperO2: Hyperoxia
PulmBundl: pulmonary bundle protocol
DVT: deep vein thrombosis
GI: Gastrointestinal.
