# Supplementary material for: Operation analysis of the tele-critical care service demonstrates value delivery, service adaptation over time, and distress among tele-providers
Source: Front Med (Lausanne). 2022 Aug 5;9:883126. doi: 10.3389/fmed.2022.883126 (PMC9388902; doi:10.3389/fmed.2022.883126)
Supplement: Supplementary file 1 [file Table_1.docx]

**Supplemental Table #1** Different hospital systems with their corresponding ICUs described by their number of beds and unit specialty.

| **Hospital** | **ICU Number** | **Number of Beds** | **Description** |
| --- | --- | --- | --- |
| **Hospital 1** | 12 | 24 | Medical ICU |
| **Hospital 2** | 13 | 14 | General ICU |
| **Hospital 3** | 14 | 6 | Long-term care facility ICU |
| **Hospital 4** | 5 | 22 | Neuro ICU |
|  | 6 | 16 | Surgical ICU |
|  | 7 | 36 | Heart & Vascular ICU |
|  | 8 | 12 | Cardiac ICU |
|  | 9 | 24 | Medical ICU |
| **Hospital 5** | 15 | 24 | General ICU |
| **Hospital 6** | 11 | 16 | Surgery & Cardiac & Neuro ICU |
|  | 10 | 13 | Medical ICU |
| **Hospital 7** | 1 | 16 | Medical ICU |
|  | 2 | 24 | Heart & Vascular ICU Cardiac ICU |
|  | 3 | 24 | Trauma & Surgical ICU |
|  | 4 | 24 | Neuro ICU |
