# Supplementary material for: Operation analysis of the tele-critical care service demonstrates value delivery, service adaptation over time, and distress among tele-providers
Source: Front Med (Lausanne). 2022 Aug 5;9:883126. doi: 10.3389/fmed.2022.883126 (PMC9388902; doi:10.3389/fmed.2022.883126)
Supplement: Supplementary file 2 [file Table_2.docx]

**Supplemental Table #2** Description and abbreviation of predetermined tasks broken done by provider type

| Staff | Task | Task definition | Abbreviation |
| --- | --- | --- | --- |
|  |  |  |  |
| eMD | Intensivist Support/Oversight | Intensivists providing or augmenting the delivery of critical care services in general | eMDSupprt |
|  | Unstable by Trends | Intensivists addressing vital signs or lab trends deemed unfavorable | Unstable |
|  | ARDS | Intensivists addressing issues related to acute distress respiratory syndrome | ARDS |
|  | Admission | Intensivists assessing newly admitted patient | ADM |
|  | Shock Support | Intensivists providing or augmenting the delivery of critical care services for patients in shock | ShockSupprt |
|  | Cardiac Arrest | Intensivists providing or augmenting the delivery of critical care services for patients in active cardiac arrest. | CPRSupprt |
|  | TTM/Post-Arrest Support | Intensivists providing or augmenting the delivery of critical care services for patients in case of ROSC after cardiac arrest. | TTMSupport |
|  | Other (TTM/Post-Arrest support, AKI,) | Intensivists providing or augmenting the delivery of critical care services not specified above | Other |
| eRN | Clinical Follow-up | eRN following up on clinical problems signaled by any of the triggers | Follow |
|  | RASS & and Sedation | eRN actively following up on addressing the adequacy of sedation protocol using PREDETERMINED PROTOCOL | RASS |
|  | Investigating Data Veracity | eRN investigating the accuracy of alarm generated by a computer system | DatVeracity |
|  | Clerical Entry Correction | eRN investigating the accuracy of alarm generated by unit staff | ClerVeracity |
|  | Additional Support | eRN rending expertise and time to the bedsite staff on otherwise not specified tasks | Additional |
|  | Impulsive or Agitated Patient | eRN assisting unit in dealing with agitated or impulsive patients | AgitPat |
|  | SBT/SAT | eRN following up on addressing readiness for initiation of spontaneous breathing trials using PREDETERMINED PROTOCOL | SBT/SAT |
|  | **Check on Continuous Infusions** | eRN providing an additional check on infusion setup on request from bedside staff | ContInf |
|  | Oversight During Procedure | eRN providing expertise during the procedure | Oversight |
|  | Compliance | eRN addressing compliance of executed orders with documentation | Compliance |
|  | Aggregated Time   - DVT - GI | eRN following up on addressing the adequacy of sedation protocol using PREDETERMINED PROTOCOL addressing the implementation of DVT & GI prophylaxis, and initiation of spontaneous breathing trials, when no intervention took place | Aggregated |
|  | Other (additional support, continuous infusion check, impulsive or agitated patient, oversight during procedure) | Intensivists providing or augmenting the delivery of critical care services not specified above | Other |
| eRT | Declining Intubated | eRT addressing worsening respiratory status in an intubated patient | DecVent |
|  | Declining Non-Intubated | eRT addressing worsening respiratory status in an intubated patient | DecnonVent |
|  | Extubation | eRT addressing issues high risk extubation screen or providing support during high-risk extubation | Extubation |
|  | Hyperoxia | eRT providing or augmenting the delivery of critical care services not specified above | HyperO2 |
|  | Pulmonary Bundle | eRT providing or augmenting the delivery of critical care services not specified above | PulmBundl |
|  | Deferring | eRT following on task assigned by bed-site staff | Defer |
|  | SBT/SAT | eRT following up on addressing readiness for initiation of spontaneous breathing trials using PREDETERMINED PROTOCOL | SBT/SAT |
|  | Compliance | eRT addressing compliance of executed orders with documentation | Compliance |
|  | ARDS | eRT addressing issues related to acute distress respiratory syndrome | ARDS |
|  | Other | eRT providing or augmenting the delivery of critical care services not specified above | Other |
