# Supplementary material for: Operation analysis of the tele-critical care service demonstrates value delivery, service adaptation over time, and distress among tele-providers
Source: Front Med (Lausanne). 2022 Aug 5;9:883126. doi: 10.3389/fmed.2022.883126 (PMC9388902; doi:10.3389/fmed.2022.883126)
Supplement: Supplementary file 3 [file Table_3.docx]

**Supplemental Table #3** The total amount of times each specialty communicated with the different groups at Penn e-Lert with bedside staff.

| **Interlocutor** | **eMD** | **eRN** | **eRT** |
| --- | --- | --- | --- |
| **PENN E-LERT** | 76 (52.24) | **776 (255.06)** | **160 (704.69)** |
| **Bedside Staff** | 968 (991.76) | 4,321 (4,841.94) | 13,922 (13,377.31) |
| **Attending/eMD** | **114 (44.08)** | **604 (201.73)** | **179 (651.19)** |
| **House Staff** | **259 (138.43)** | 570 (633.53) | 1,988 (2045.04) |
| **APP/eAPP** | **246 (103.84)** | **318 (475.20)** | 1,549 (1533.96) |
| **RN/eRN** | **206 (113.57)** | **1,559 (519.73)** | **546 (1677.70)** |
| **RT/eRT** | **117 (542.08)** | **1,260 (2480.81)** | **9,654 (8008.11)** |

The expected number of communications in parentheses.
