# Supplementary material for: Operation analysis of the tele-critical care service demonstrates value delivery, service adaptation over time, and distress among tele-providers
Source: Front Med (Lausanne). 2022 Aug 5;9:883126. doi: 10.3389/fmed.2022.883126 (PMC9388902; doi:10.3389/fmed.2022.883126)
Supplement: Supplementary file 4 [file Table_4.docx]

**Supplemental Table #4** The overall responses of each specialty to intervention requests.

|  | **eMD** | **eRN** | **eRT** |
| --- | --- | --- | --- |
| **Accepted** | 557 (537.92) | 2,609 (2670.88) | 6,449 (6406.20) |
| **Acknowledged** | 81 (95.05) | 528 (471.95) | 1,090 (1131.99) |
| **Rejected** | 22 (27.02) | 140 (134.17) | **321 (321.81)** |

The expected number of communications in parentheses.
