# Supplementary figures and images for: Operation analysis of the tele-critical care service demonstrates value delivery, service adaptation over time, and distress among tele-providers
Source: Front Med (Lausanne). 2022 Aug 5;9:883126. doi: 10.3389/fmed.2022.883126 (PMC9388902; doi:10.3389/fmed.2022.883126)

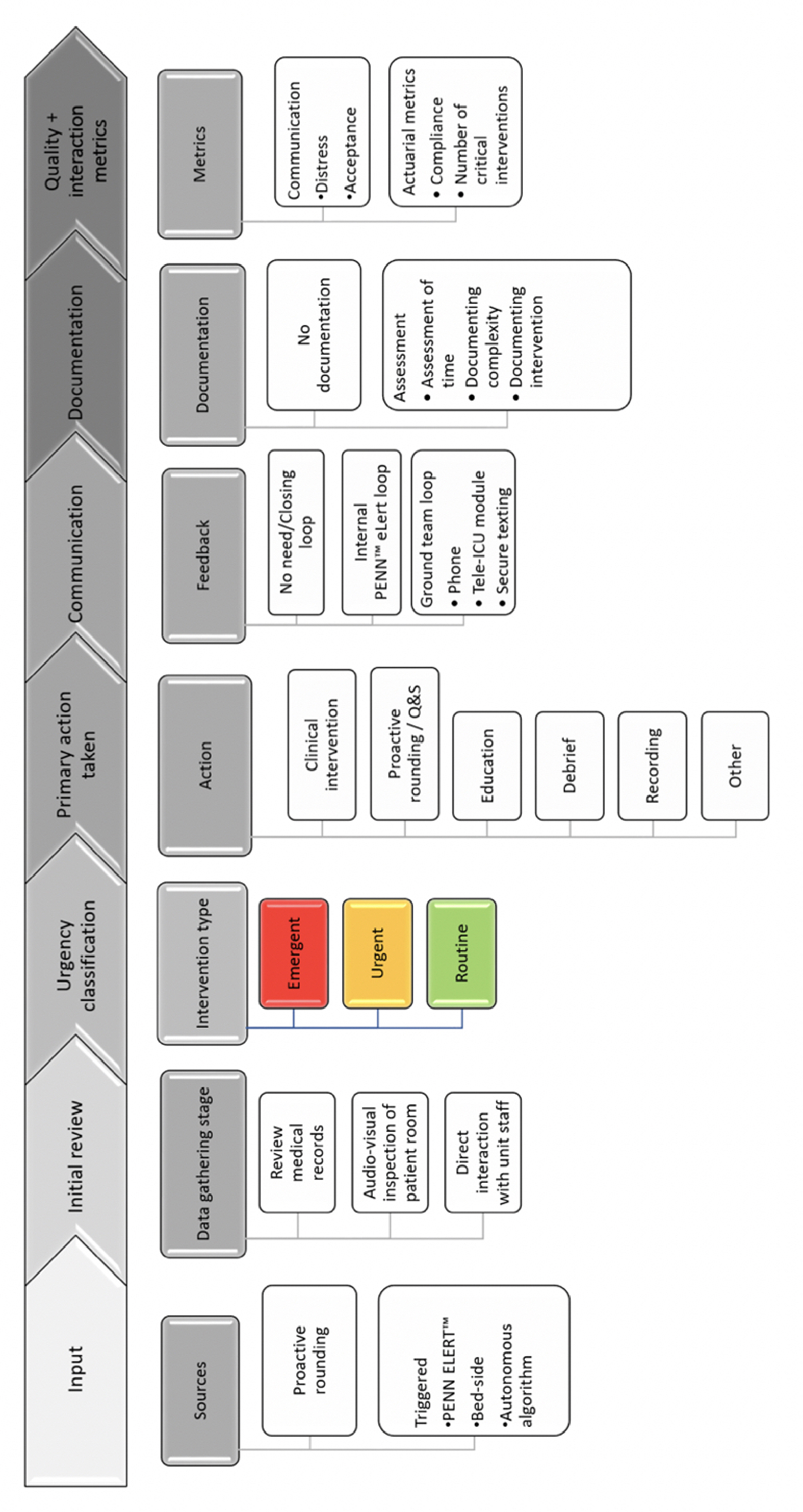

Supplement: Supplementary file 5 [file Image_1.JPEG]

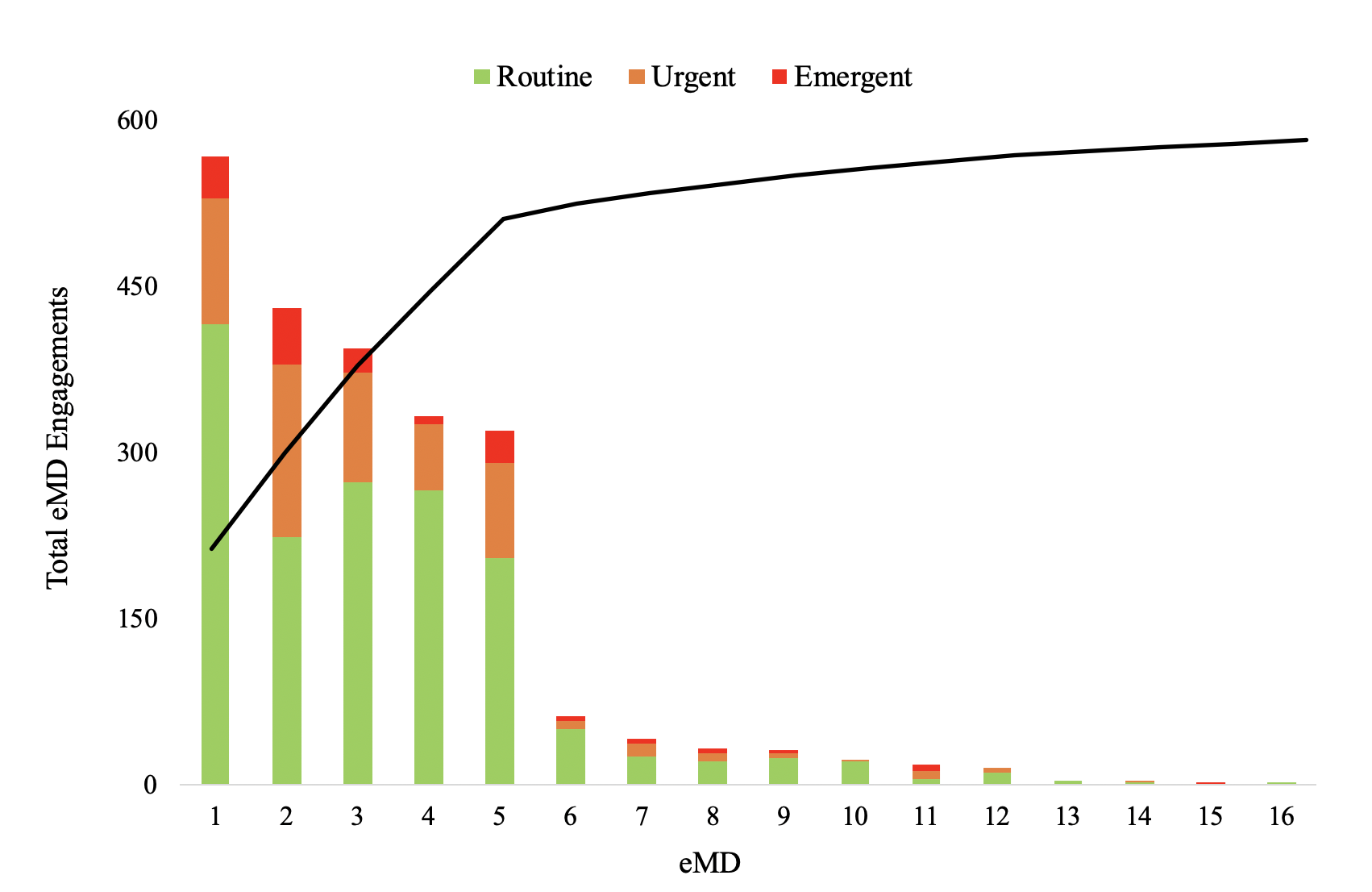

Supplement: Supplementary file 6 [file Image_2.PNG]

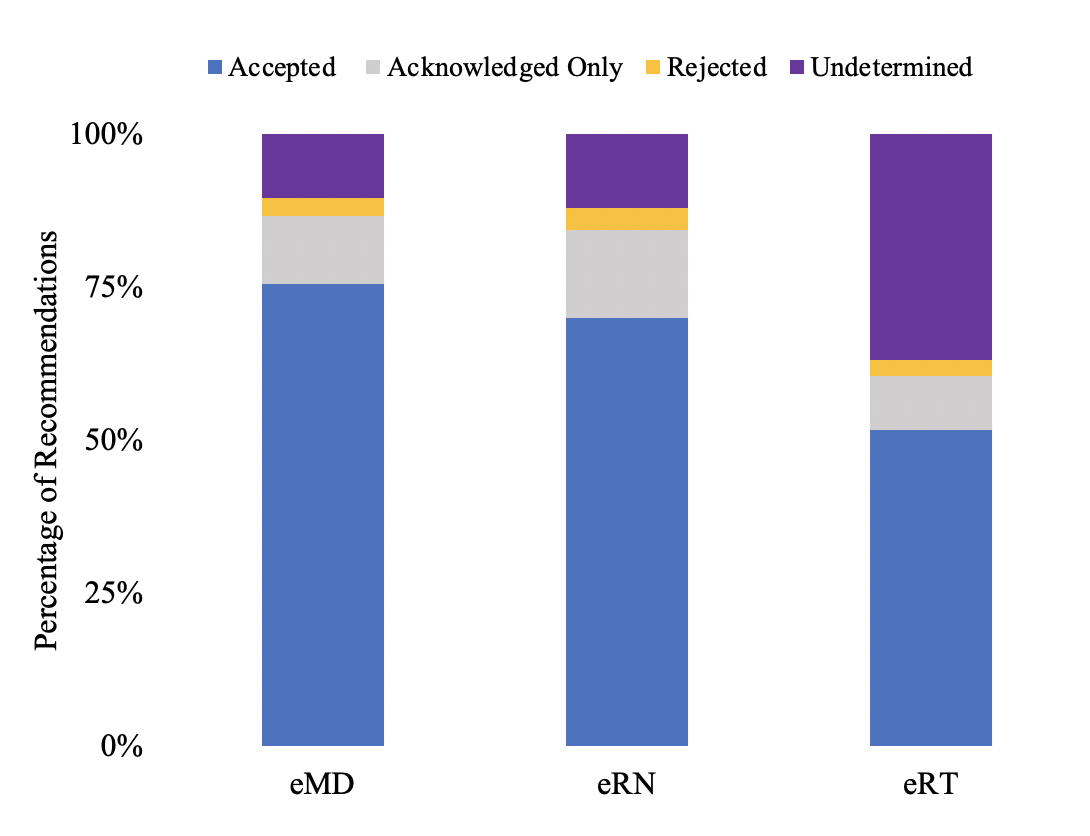

Supplement: Supplementary file 7 [file Image_3.PNG]

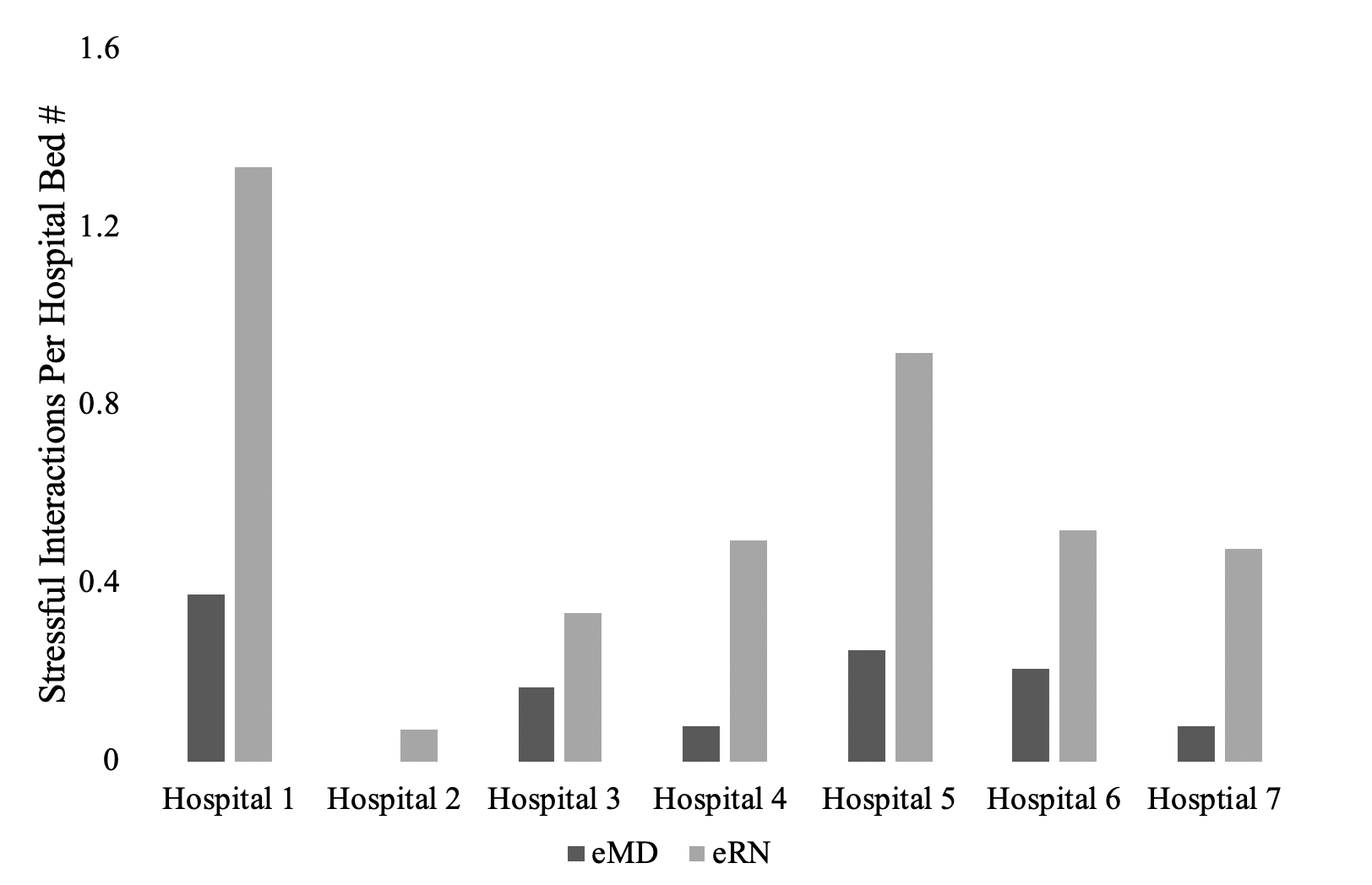

Supplement: Supplementary file 8 [file Image_4.PNG]

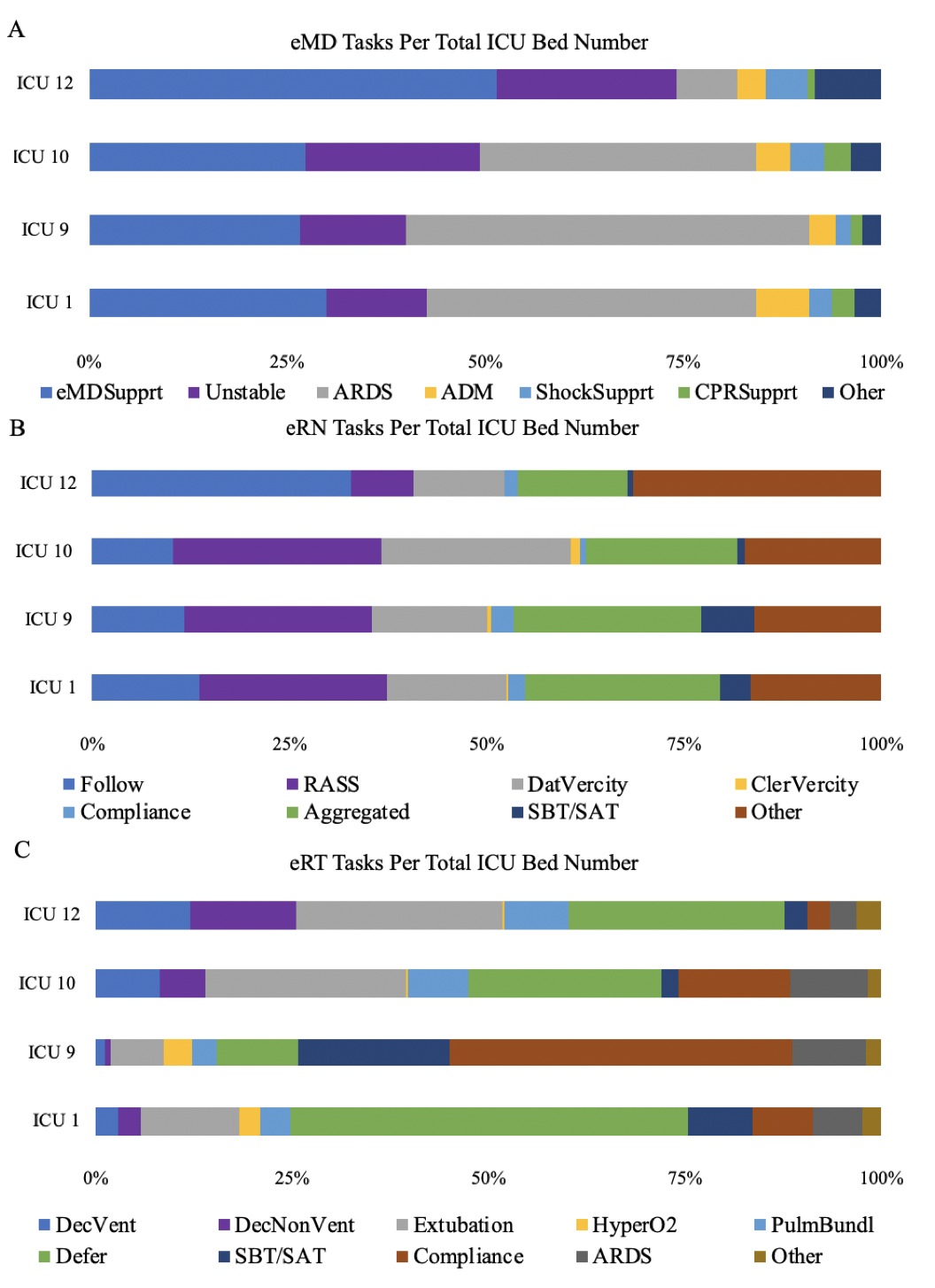

Supplement: Supplementary file 9 [file Image_5.PNG]

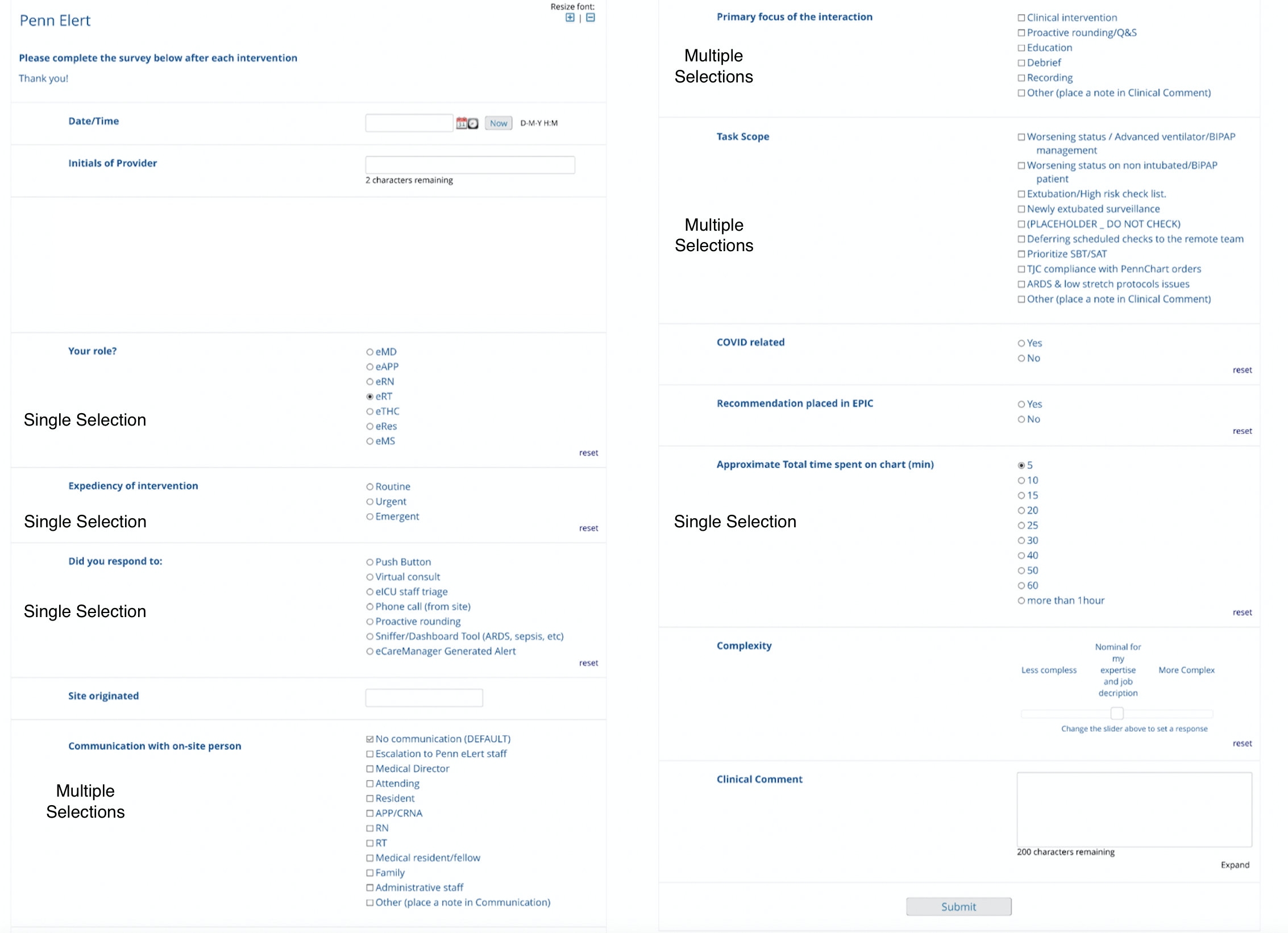

Supplement: Supplementary file 10 [file Image_6.PNG]
